# Bioemulsifiers are not biosurfactants and require different screening approaches

**DOI:** 10.3389/fmicb.2015.00245

**Published:** 2015-04-07

**Authors:** Chibuzo Uzoigwe, J. Grant Burgess, Christopher J. Ennis, Pattanathu K. S. M. Rahman

**Affiliations:** ^1^Technology Futures Institute, School of Science and Engineering, Teesside UniversityMiddlesbrough, UK; ^2^School of Marine Science and Technology, Newcastle UniversityNewcastle Upon Tyne, UK

**Keywords:** biosurfactants, bioemulsifiers, rhamnolipids, lipoproteins, lipopeptides, sophorolipids

The terms biosurfactant and bioemulsifier have often been used interchangeably to describe surface active biomolecules. However, it is important to note that there are marked differences between them especially based on their physico-chemical properties and physiological roles. Although bioemulsifiers and biosurfactants are both amphiphilic in nature and are produced by a wide range of microorganisms, each exhibit characteristic roles in nature. These microbial surfactants have recently received increased scientific attention due to their unique characteristics relative to chemically derived surfactants. Their unique features include; non-toxicity, biodegradability, biocompatibility, efficiency at low concentrations and their synthesis from natural substrates under mild environmental conditions.

## The physiological roles of biosurfactants and bioemulsifers are based on their physicochemical properties

The chemical composition of biosurfactants and bioemulsifiers is different and this may contribute to their specific roles in nature and biotechnological applications. Biosurfactants are generally low molecular weight microbial products composed of sugars, amino acids, fatty acids and functional groups such as carboxylic acids. The glycolipids (rhamnolipids, sophorolipids, trehalose lipids) consist of different sugars linked to β-hydroxy fatty acids while lipopeptides (surfactin, iturin, fengycin) consist of cycloheptapeptides with amino acids linked to fatty acids of different chain lengths. These molecules are amphiphilic in nature and this property allows them to dissolve in both polar and non-polar solvents (Perfumo et al., 2009; Satpute et al., 2010; Smyth et al., 2010a,b). Biosurfactants are known for their excellent surface activity which involves lowering the surface and interfacial tension between different phases (liquid-air, liquid-liquid, and liquid-solid); possessing a low critical micelle concentration (CMC) and formation of stable emulsions. The ability to lower surface and interfacial tension is by adsorption of biosurfactant onto the different phases causing more interaction and mixing of dissimilar phases. The CMC is the minimum concentration of biosurfactant required to yield the minimum surface tension in water and form micelles. They can act as wetting, foaming and solubilizing agents in different industrial processes. In oil polluted environments (solid or liquid), biosurfactants can enhance the effective dispersion and bioavailability of hydrophobic pollutants for microbial access and degradation by the process of micelle solubilization. They have the ability to mobilize hydrophobic molecules bound on solid substrata increasing the flow rate. Biosurfactants produced in a growth associated manner (trehalose lipids) confer increased cell surface hydrophobicity on the producing organism. Cell surface hydrophobicity is essential for easy access and subsequent uptake of hydrophobic substrates by microbial cells (Perfumo et al., 2009; Satpute et al., 2010). These biomolecules are therefore suitable agents for different bioremediation technologies.

Bioemulsifiers are higher in molecular weight than biosurfactants as they are complex mixtures of heteropolysaccharides, lipopolysaccharides, lipoproteins and proteins (Perfumo et al., 2009; Smyth et al., 2010a; Sekhon-Randhawa, 2014). They are also known as high molecular weight biopolymers or exopolysaccharides. Similar to biosurfactants, these molecules can efficiently emulsify two immiscible liquids such as hydrocarbons or other hydrophobic substrates even at low concentrations but in contrast are less effective at surface tension reduction. Therefore, they can be said to possess only emulsifying activity and not surface activity. They are also involved in solubilization of poorly-soluble substrates, thus increasing their access and availability for biodegradation. However, in an oil polluted environment, these molecules play a specific role of binding tightly to dispersed hydrocarbons and oils preventing them from merging together. This process is known as stabilization of emulsion and has been attributed to the high number of reactive groups exposed in their structures. Bioemulsifiers are able to stabilize emulsions by increasing their kinetic stability and this property has increased their usefulness in the cosmetics, food, pharmaceutical and petroleum industries. Reports have shown that the efficient emulsifying activity of bioemulsifiers is a function of their chemical composition (Calvo et al., 2009; Monteiro et al., 2010). According to Willumsen and Karlson (1997), surface active biomolecules are categorized into surfactants and emulsifiers, while surfactants play the role of surface tension reduction, emulsifiers are involved in formation and stabilization of emulsions. However, some biomolecules possess both surfactant and emulsifying properties which contributes to their unique functions and broad industrial uses.

The combination of polysaccharide, fatty acid and protein components in bioemulsifiers confers upon them better emulsifying potential and ability to stabilize emulsions. It is also important to note that some efficient bioemulsifiers consists of only polysaccharides and proteins. Emulsan is a lipopolysaccaharide bioemulsifier with a molecular weight of 1000 kDa produced by *Acinetobacter calcoaceticus* RAG-1. It is one of the most widely studied emulsifiers from bacteria. In pure form, emulsan shows emulsifying activity at low concentrations (0.01–0.001%). It increases the bioavailability of poorly soluble substrates in aqueous environments for microbial access and degradation by coating the hydrophobic substrate to form a minicapsule. The producing bacterium can also have direct access to hydrophobic substrates but the emulsifying activity is exhibited by secreted emulsan. However, this emulsifier can efficiently emulsify mixtures of aliphatic and aromatic hydrocarbons in balanced proportions but cannot emulsify their pure forms. The emulsifying activities of emulsan are therefore attributed to its fatty acid components which act as multiple sites for binding different hydrophobic phases (Choi et al., 1996; Ron and Rosenberg, 2001).

A bioemulsifier also known as emulsan produced by *Acinetobacter calcoaceticus* BD4 has been reported. BD4 Emulsan consists of a polysaccharide-protein complex without a lipid moiety thereby varying from RAG-1 Emulsan. BD4 emulsan exhibited its optimum emulsification of different hydrocarbons when the two components (polysaccharides and proteins) were mixed together but had no emulsifying activity when these components existed in separate forms (Kaplan and Roseberg, 1985). Kaplan et al. (1987) also explained the mechanism of emulsifying activity by the protein-polysaccharide mixture. The protein being the hydrophobic part binds the hydrocarbons in a reversible form while the polysaccharide attaches to the protein to produce a stable oil-in-water emulsion. Lukondeh et al. (2003) also described bioemulsifiers as amphiphilic, with the polysaccharide polymer as the hydrophilic part covalently attached to the protein hydrophobic part.

Furthermore, alasan is an anionic alanine-containing bioemulsifier produced by *A. radioresistens* KA53. This bioemulsifier is a complex of alanine, polysaccharides and proteins with a molecular mass of 1 MDa. It exists as cell-bound and secreted proteins and can efficiently emulsify a variety of hydrocarbons including; long chains, alkanes, aromatics, poly aromatic hydrocarbons (PAHs), paraffins and crude oils. Alasan can facilitate solubilization of PAH by aggregating them into oligomer molecules and this mechanism increases their solubility by 20-fold, thereby speeding up biodegradation. The protein components in the alasan molecule have been associated with its ability to emulsify various hydrocarbons unlike emulsan. This is a 45 kDa protein with highly hydrophobic regions folded over in loops contributing to emulsification and solubilization activity of alasan (Navon-Venezia et al., 1995). The contribution of protein components to the emulsifying potential of bioemulsifiers have also been reported in the literature. Sar and Rosenberg (1983) have also demonstrated that the protein content of bioemulsifier plays an important role in the emulsification activity.

Mannoproteins are glycoproteins extracted from the cell walls of many yeasts. These molecules are classified as structural and enzymatic mannoproteins depending on their chemical compositions and specific functions in living systems. Structural mannoproteins are the most abundant and are composed of a small protein portion linked to a greater carbohydrate portion (mannopyranosyl) while enzymatic mannoproteins have more protein moieties in their structures. These molecules are not only effective emulsifiers but have been associated with stimulation of host immunity by activating immune cells and proteins as well as triggering the production of antibodies (Casanova et al., 1992; Oliveira et al., 2009). A mannoprotein bioemulsifier from *Kluyveromyces marxianus* has been reported to form a 3-month old stable emulsion in corn oil (Lukondeh et al., 2003). Alcantara et al. (2014) reported a mannoprotein bioemulsifier from *Saccharomyces cerevisiae* 2031 consisting of 77% carbohydrate and 23% protein. A bioemulsifier with 53% protein, 42% polysaccharide and only 2% lipid has been reported from *Acinetobacter* sp. by Jagtap et al. (2010). These mannoprotein emulsifiers were capable of forming stable emulsions with different hydrocarbons, organic solvents and waste oils, suggesting their applications as cleaning agents.

Chemical components such as uronic acid have also been associated with the emulsifying capability of bioemulsifiers. *Halomonas eurihalina* produces an exopolysaacharide (EPS) that can efficiently emulsify hydrocarbons. This EPS is rich in uronic acids containing less carbohydrate and protein components. The uronic acid has been associated with the ability of EPS to emulsify and detoxify hydrocarbons (Martínez-Checa et al., 2002). Jain et al. (2013) also reported on a bioemulsifier with a molecular weight of 2716 kDa, consisting of mainly total sugars, uronic acids and proteins produced by *Klebsiella* sp. On a general note bioemulsifiers have been associated with a number of potential applications including: remediation of oil polluted water and soil; enhanced oil recovery and clean-up of oil contaminated vessels and machineries; heavy metal removal (Monteiro et al., 2010; Zheng et al., 2012; Panjiar et al., 2014); formation of stable emulsions in food and cosmetics industries (Campos et al., 2014); and therapeutic activities (antibacterial, antifungal, pesticidal and herbicidal agents) (Ahmed and Hassan, 2013). The physico-chemical properties of bioemulsifiers and biosurfactants are presented in Table 1.

**Table 1:** **Physicochemical properties of biosurfactants and bioemulsifiers**.

**Biosurfactant**	**Class**	**Microbial origin**	**Physicochemical properties**	**Physiological roles**	**References**
Low molecular weight	Rhamnolipids	*Pseudomonas aeruginosa DS10-129*	One or two rhamnose sugars linked to 3-hydroxydecanoic acid.	Bioremediation technology	Rahman et al., 2002
Glycolipid			ST- 28 mN/m		
			EI24- 53–73%		
	Sophorolipids	*Candida bombicola*	Disaccharide sophoroses (2-O-β-D-glucopyranosyl-D-glucopyranose) linked to fatty acids.	-Detergent additive for enhanced performance and stain removal	Develter and Lauryssen, 2010; Joshi-Navare et al., 2013
		*Candida tropicalis*	ST- 32.1–34.2 mN/m	-Hard surface cleaning, Antibacterial activity.	
	Trehaloselipid	*Rhodococcus wratislaviensis BN38*	Non-reducing disaccharide with two glucose units linked in an α,α-1,1-glycosidic linkage	Bioremediation of polluted sites, Antitumor activity	Tuleva et al., 2008; Christova et al., 2014
		*Norcardia farcinica BN26*	ST-24.4 mN/m		
			EI- 23-70%		
Lipopeptides	Surfactin	*Bacillus subtilis K1*	Heptacyclic depipeptides consisting of two acidic amino acids, four hydrophobic amino acids and C_13-17_ β-hydroxyfatty acids	-Enhanced oil recovery	Ongena and Jacques, 2007; Varadavenkatesan and Ramachandra, 2013; Pathak and Keharia, 2014
		*Bacillus siamensis*	ST- 22–27.9 mN/m	-Antibacterial	
				-Antiviral	
				-Antimycoplasma	
				-Antitumoral	
				-Anticoagulant	
				-Enzyme inhibition	
	Iturin	*Bacillus subtilis K1*	Cycloheptapeptide with seven amino acids and C13-16 β-amino fatty acids	-Antifungal	Arrebola et al., 2010; Pathak and Keharia, 2014
		*Bacillus amylofaciens*	ST-30–37 mN/m	-Biopesticides	
			EI- 32–66%		
	Fengycin	*Bacillus subtilis*	Cyclic deca-depsipeptides with C14-21 β-hydroxyfatty acid and 10 amino acids	-Strong fungitoxic agent against filamentous fungi, Immunomodulating activities	Arrebola et al., 2010; Pathak and Keharia, 2014
High molecular weight- Bioemulsifiers	RAG-1 Emulsan	*Acinetobacter* sp. *ATCC 31012 (RAG-1)*	Lipopolysaccharides. Lipid moiety (unsaturated fatty acid of C10-18) Polysaccharide moiety (D-galactosamine, D-galactosaminuronic acid, di-amino-6-deoxy-D-glucose)	-Increase surface area and bioavailiability of poorly-soluble substrates	Choi et al., 1996; Ron and Rosenberg, 2001
				-Binding to toxic heavy metals	
	BD4 Emulsan	*Acinetobacter calcoaceticus BD4 13*	Protein-polysaccharide complex.	Stabilizes oil-in-water emulsions	Kaplan and Roseberg, 1985; Kaplan et al., 1987
			Polysaccharide moiety (repeating heptasaccharides of L-rhamnose, D-glucouronic acid, D-mannose)		
	Alasan	*Acientobacter radioresistens KA53*	Alanine-containing polysaccharide and proteins	Emulsification and solubilization activity	Navon-Venezia et al., 1995; Walzer et al., 2006
	Mannoproteins	*Saccharomyces cerevisiae*	Polysaacharide and proteins	Formation of stable emulsion with hydrophobic substrates Stimulation of immune system	Casanova et al., 1992; Lukondeh et al., 2003
		*Kluyveromyces marxianus*			
	Uronic acid bioemulsifiers	*Halomonas eurihalina Klebsiella* sp.	Polysaccharides-proteins-uronic acids	Emulsification and detoxification of hydrocarbons	Martínez-Checa et al., 2002; Jain et al., 2013

At this point, it is important to note that the ability to reduce surface and interfacial tension stands as the distinctive contrast between biosurfactant and bioemulsifiers. These molecules can both form stable emulsions but it is still unclear why bioemulsifiers do not show significant changes in surface/interfacial tension between different phases (liquid-air, liquid-liquid, liquid-solid). This outstanding contrast between biosurfactants and bioemulsifiers is especially important for accurate screening and identification procedures from microbial culture broths.

Screening for detection of biological surfactants in culture media has often been based on the measurement of surface and interfacial tension. Other methods include drop collapse, oil displacement, haemolysis tests and use of the emulsification index E_24_ (Cooper and Goldenberg, 1987), emulsification activity (Rahman et al., 2002, 2010; Satpute et al., 2008), and bacterial adhesion to hydrocarbon assay (BATH) also known as cell surface hydrophobicity (Rosenberg et al., 1980). According to Satpute et al. (2008), these methods are insufficient for the identification and differentiation of bioemulsifiers from biosurfactants. This is due to the fact that bioemulsifiers are best known for emulsification of liquids without significant changes in surface/interfacial tension of their growth medium or between different phases. In addition, experimental reports have shown that surface tension measurements and emulsification index/activity screening methods do not correlate. These methods have often resulted in elimination of bioemulsifiers since they do not exhibit significant changes in surface/interfacial tension and may give negative results during screening tests (Ellaiah et al., 2002). Emulsification index E_24_ and emulsification activity are screening tests for measuring the emulsification capacity of any surface active molecule with different hydrocarbons (Jagtap et al., 2010). BATH is an indirect screening method to detect cell-bound emulsifying agents produced by microorganisms. Cell-bound surface active agents are important in hydrocarbon assimilation during biodegradation and bioremediation of polluted environment. When microorganisms show low surface hydrophobicity it is an indication that emulsifying agents have been released extracellularly into the production media.

Using the emulsification index E_24_ test, Viramontes-Ramos et al. (2010) identified six isolates that could efficiently emulsify different hydrocarbons (more than 50% against diesel, decane, kerosene and motor oil) without showing a significant reduction in surface tension of their culture broths. The emulsifying potential of surface active compounds from different yeasts showing no reduction in surface tension, has been evaluated using the emulsification index test (Amaral et al., 2006; Monteiro et al., 2010). Toledo et al. (2008) reported the growth and production of exopolymers by three bacteria, *Bacillus subtilis* 28, *Alcaligenes faecalis* 212, and *Enterobacter* sp. 214 on glucose media amended with different hydrocarbons. These biopolymers exhibited high emulsifying activity but without reduction in surface tension of their culture media. *Candida tropicalis* grown on n-hexadecane produced an extracellular emulsifier with surface tension of 49.5 mN/m but was capable of emulsifying various hydrocarbons including aromatic hydrocarbons (Singh and Desai, 1989). Souza et al. (2012) identified a potent bioemulsifier produced by *Yarrowia lipolytica* which exhibited high emulsification values with hydrocarbons, with no reduction in surface tension.

## Concluding remarks

A number of literature reports have regarded all microbial surfactants as biosurfactants even when bioemulsifiers have been identified. These two types of surfactants are closely related especially in their ability to form stable emulsions. However, screening methods for identification of microbial surfactants based on surface tension reduction are bound to eliminate producers of bioemulsifiers and retain producers of biosurfactants. Therefore, the opinion of authors in this review is that bioemulsifiers are not biosurfactants (they both emulsify, but only biosurfactants have the surfactant effect of reducing surface tension) and current research toward identification should be based on a broad spectrum of tests and not only on surface tension measurements, which are often used as primary tests. In addition, there is minimal research on the discovery and characterization of new emulsifiers from microorganisms due to the lack of a clear picture of the distinguishing features between biosurfactants and bioemulsifiers. Biosurfactants and bioemulsifiers are both unique microbial products showing advantageous features and may become future substitutes for chemically produced ones. We have noted that bioemulsifiers have often been erroneously eliminated or mis-identified in the past but, since these molecules have great potential for green technology, carefully designed screening methods will be an essential step toward the discovery of novel microbial emulsifiers.

### Conflict of interest statement

The authors declare that the research was conducted in the absence of any commercial or financial relationships that could be construed as a potential conflict of interest.
